# How children learn complex environmental concepts through digital storytelling: divergent pathways to knowledge and stewardship intention

**DOI:** 10.3389/fpsyg.2026.1893526

**Published:** 2026-07-31

**Authors:** Xin Bian, Andre Brown, Bruno Marques

**Affiliations:** 1 School of Art and Design, Shandong Women’s University, Jinan, China; 2 Wellington School of Architecture, Victoria University of Wellington, Wellington, New Zealand

**Keywords:** cognitive load, digital storytelling, multimedia learning, primary school students, sustainability education

## Abstract

Digital storytelling is increasingly employed in sustainability education to communicate complex place-based environmental issues. However, it remains unclear how primary school students encode concept-dense content during brief narrative viewing, and how such encounters relate to content knowledge (CK) and place-based sustainability stewardship intention (PESI). Using the industrial heritage of the Dagushan Iron Mine as a place-based context, this classroom follow-up study examined whether CK and PESI showed different patterns of association during brief narrative viewing. In the main phase, 452 students in Years 1–6 watched a five-minute animation and completed a seven-item CK test plus experience scales assessing experiential dimensions including interest, engagement, emotional response, audiovisual experience, educational value, and PESI. Associations were analyzed using multiple regression and structural equation modelling. CK was measured at baseline and immediately post-viewing, with a supplementary follow-up conducted 2 weeks later assessing CK and retrospective perceived cognitive load. Results showed selective uptake under brief viewing. Interest positively predicted CK, whereas audiovisual experience and educational value showed inverse associations with CK in the structural model, consistent with a processing-demand account, with additional supporting evidence from retrospective perceived cognitive-load findings at follow-up. CK was not associated with PESI; instead, PESI was linked to grade level and experiential appraisals. At follow-up, higher retrospective perceived extraneous cognitive load was associated with lower CK. Overall, brief narrative animations can support early engagement with complex environmental content, while conceptual understanding and stewardship-oriented intention may emerge through partially distinct learning processes. For classroom practice, the findings suggest that brief narrative animations are effective for introducing complex environmental topics and stimulating students’ initial engagement, but should be supplemented with instructional scaffolding, guided explanation, and structured follow-up activities to support deeper conceptual understanding.

## 1 Introduction

Contemporary children frequently encounter complex social, historical, and environmental issues within both educational curricula and daily life. These subjects often encompass multiple temporal scales, extensive causal chains, and abstract value judgments, which impose significant cognitive demands on primary school students. These learners are still developing capacities such as working memory and integrative reasoning (Case, 1992). From an environmental and sustainability education perspective, such complexity is not only informational but interpretive: learners must make sense of human–environment relations, trace consequences over time, and form early responsibility-oriented judgements (Peretz, 2025). Industrial heritage serves as a prototypical example of this conceptual complexity because post-industrial landscapes often combine technological process understanding with environmental consequence and restoration narratives (Bian et al., 2026). A prototypical case is the Dagushan Iron Mine in Anshan, China, a century-old open-pit iron mine that illustrates the long-term interaction between industrial development, environmental change, and ongoing ecological restoration, making it an appropriate place-based context for sustainability education. To understand the processes of extraction, processing, and transportation, together with their associated social and environmental impacts, students must comprehend extended time sequences and the interconnected processes linking industrial activities, environmental change, and ecological restoration while also interpreting value-laden meanings (Cocal-Smith et al., 2023). In particular, they are asked to connect human actions with environmental change and the possibility of ecological restoration, which positions industrial heritage as a place-based entry point to sustainability-relevant meaning making rather than as historical knowledge alone (Hernandez Gonzalez, 2023). For younger students, these cognitive requirements may exceed their current processing capabilities, highlighting an important challenge for environmental and sustainability education as well as the learning sciences: understanding how children develop both content knowledge and stewardship-oriented intentions when complex content is presented through brief narrative formats under time constraints (Mayer, 2024).

Digital storytelling has gained considerable traction in educational research due to its ability to present complex content in visually structured and narratively coherent ways (Bian et al., 2025b). Grounded in multimedia learning theory, research suggests that effective alignment of verbal and visual information, along with controlled extraneous cognitive load, enables animations to facilitate learning under optimal design conditions (Mayer, 2024). Meta-analytic studies also show that dynamic visualizations are effective for depicting temporal changes and causal mechanisms when the pacing and signaling are tailored to match learners’ processing abilities (Castro-Alonso et al., 2019). However, highly interactive learning environments, such as educational games and virtual reality, while engaging, often introduce additional extraneous loads due to navigation demands, interface manipulation, and multimodal stimulation, particularly for younger learners (Makransky and Petersen, 2021). For environmental and sustainability education topics that involve long causal chains and time-delayed consequences, brief narrative animations are increasingly used as accessible classroom resources, yet they also intensify the challenge of encoding concept-dense information within limited processing time (Otto, 2025). In this context, low-interactivity narrative animations offer a controlled yet ecologically valid approach to explore how children process concept-dense information under time constraints. Environmental education research has previously used delayed assessments to examine children’s recall from environmental education videos, reporting sustained motivation and comparable recall patterns across learner groups when videos are paired with pedagogical support (Viteri et al., 2013). However, in reception-focused, time-constrained viewing episodes, such work has rarely modelled how multiple experiential appraisals relate simultaneously to both objective knowledge performance and value-oriented, place-based stewardship intentions in a single brief narrative episode.

Within such time-constrained narrative viewing, objective knowledge performance is shaped by processing demands, whereas learners’ subjective experiences provide complementary insights into their immediate evaluative responses. Previous research has identified several experiential dimensions pertinent to video-based learning—such as interest, emotional response, perceived comprehension, audiovisual quality, and perceived educational value—that are intricately linked to attention allocation, motivational regulation, and metacognitive monitoring (Fredricks et al., 2004). Developmental studies have also demonstrated that working memory capacity, strategy use, and evaluative judgment systematically evolve across grade levels, influencing learners’ interpretations of narrative content and their assessments of its broader relevance (Boetje et al., 2024). In environmental and sustainability education, these developmental differences may be especially salient for value-oriented outcomes, because older students are typically better able to contextualize place-based narratives, weigh consequences, and articulate responsibility-oriented positions (Soysal et al., 2026). These findings suggest that both experiential appraisals and developmental differences may influence children’s responses in various ways. Accordingly, knowledge acquisition and place-based sustainability stewardship intentions may show distinct patterns of association in brief, concept-dense viewing episodes. In relation to Sustainable Development Goal 4 (Quality Education) (UNESCO, 2016), this study contributes evidence on how learning quality in primary environmental education should be understood and evaluated, by examining how brief digital storytelling supports children’s engagement with complex place-based environmental content and their early stewardship-oriented intentions. In this study, Place-based Sustainability Stewardship Intention (PESI) is conceptualized as an early stewardship-oriented intention reflecting children’s willingness to care for, value, and support the sustainable future of a specific place, rather than as a direct behavioral intention. This conceptualization is consistent with stewardship perspectives in environmental and sustainability education, where value-oriented intentions are regarded as an important precursor to later pro-environmental behavior.

Despite the expanding research on digital storytelling, three significant gaps persist. Firstly, few studies have explored, within a single instructional intervention, how multiple experiential dimensions correlate with Content Knowledge (CK) and value-oriented support intentions, while clearly accounting for the constraints on knowledge performance imposed by time limitations. Secondly, the application of digital storytelling in primary-level industrial heritage education is infrequent, and systematic comparisons across grade levels are rare, despite the considerable conceptual complexity and socio-cultural specificity of industrial heritage topics. Thirdly, the relationship between knowledge acquisition and support intentions remains ambiguous in contexts of short-duration narratives. While prior work often reports only modest links between knowledge and intention in environmental and heritage education, it remains unclear whether similar patterns emerge when attention, affect, and pacing shape what is encoded and valued in brief narrative viewing.

To address these gaps, this study poses three interrelated research questions concerning (RQ1) changes in content knowledge over time, (RQ2) the associations between experiential dimensions and content knowledge, and (RQ3) the associations between content knowledge and Place-based Sustainability Stewardship Intention (PESI).

Methodologically, this study employs a classroom-based investigation with repeated knowledge assessments and a delayed follow-up. Students completed the CK test before viewing the animation (T0), immediately after viewing (T1), and again 2 weeks later (T2), at which point cognitive load was also measured. Experiential perception scales and PESI were collected immediately after viewing. Conceptually, short-form narrative animation is considered a processing-limited learning episode in which two outcome domains can emerge in parallel: (a) cognitive acquisition, which is expected to be constrained by processing demands under time limits; (b) value-oriented intention formation, which is expected to draw more heavily on experiential appraisal and developmental interpretation. Using data from 452 students in Years 1 to 6, we tested whether these outcomes show non-synchronous patterns of association, and whether the delayed cognitive-load evidence provides a coherent processing-demand account of knowledge performance under time constraints.

Building on this framework, the study makes three contributions. First, it advances the parallel-outcome perspective by demonstrating that content knowledge and value-oriented intentions can develop simultaneously in short narrative learning episodes without following a single, sequential pathway. Second, methodologically, it illustrates how delayed cognitive load evidence can elucidate uneven learning patterns under time constraints, rather than attributing these differences to design flaws. Third, empirically, it broadens classroom-based digital storytelling research to encompass primary-level industrial heritage education, and it underscores grade-related differences in the formation of value-oriented intentions along with selective conceptual uptake.

## 2 Literature review

Digital storytelling for environmental and sustainability education can be situated within the broader field of Education for Sustainable Development (ESD), which emphasizes the development of transversal sustainability competences, including systems thinking, value formation, and responsible action across diverse educational contexts (Bianchi et al., 2022). Within this broader perspective, digital storytelling has increasingly been recognized as a pedagogical approach for supporting learners’ understanding of complex sustainability issues by integrating narrative, visualization, and place-based learning experiences (Fadhil and Al-Alwan, 2026). Industrial heritage represents one such place-based context through which digital storytelling can simultaneously support conceptual understanding and value-oriented learning (Bian et al., 2025a). Similar narrative approaches have also been applied to fostering children’s understanding of local natural and cultural heritage (Tzima et al., 2020) and cultivating cross-cultural understanding and global sustainability awareness among students (Modi et al., 2024), highlighting the broad applicability of digital storytelling as a pedagogical approach across diverse sustainability education contexts.

### 2.1 Multimedia learning foundations for narrative animation

According to multimedia learning research, understanding is constructed through the coordination of verbal and visual information under limited working memory capacity (Hill et al., 2016). As revealed by the Cognitive Theory of Multimedia Learning (CTML), learners actively select, organize, and integrate information from both auditory verbal and visual pictorial channels, which are constrained by capacity limitations (Mayer, 2024). Cognitive Load Theory (CLT) distinguishes between intrinsic load, which arises from task complexity, and extraneous load, which results from suboptimal presentation, emphasizing the crucial role of instructional design in managing cognitive processing demands (Sweller, 2020). For dense conceptual materials, principles such as coherence, signalling, and segmenting are recommended to minimize unnecessary processing and enhance comprehension in animated and video-based instruction (de Koning et al., 2007).

In this theoretical framework, dynamic visualizations are often deemed beneficial for depicting temporal changes, causal mechanisms, and spatial transformations. Meta-analytic evidence indicates that animations and other dynamic representations can provide small to moderate learning advantages over static formats, especially when narration, pacing, and visual transitions are tailored to learners’ processing capacities (Castro-Alonso et al., 2019). However, the effectiveness of these benefits is highly context dependent. Rapid presentation or compression of information into a brief duration can lead to transient information effects, where crucial elements vanish before they can be adequately encoded and integrated, thereby increasing extraneous cognitive load (Kalyuga, 2011). Furthermore, visually complex or emotionally charged scenes may draw attention to superficial features rather than to structurally important relationships, a problem that becomes more pronounced in time-constrained classroom settings (Loderer et al., 2020). Accordingly, in environmental education contexts, delayed assessments of recall have been used to evaluate what children retain from educational videos beyond immediate viewing, providing a classroom-relevant precedent for examining the durability of learning under authentic time constraints (Viteri et al., 2013).

Industrial heritage learning materials are characterized by a strong historical context and complex multi-step causal structures, including processes of extraction, transportation, industrial transformation, and associated social and environmental changes (Cocal-Smith et al., 2023). As indicated by previous research, such materials pose significant conceptual challenges for students and visitors, particularly when explanations require integration across temporal and disciplinary boundaries (Bian et al., 2025a). In principle, low-interactivity narrative animation offers a controlled method for visualizing temporal sequences and causal relationships while reducing additional navigation demands that could distract attention. However, in contexts where narrative animations are conceptually dense and time-constrained, cognitive load provides a parsimonious explanation for the variability in knowledge performance, while experiential appraisals offer valuable insights into learners’ immediate perceptions and the construction of value-oriented meanings. In environmental and sustainability education, place-based approaches treat local landscapes as meaningful learning contexts that can foster connections to place and responsibility-oriented orientations, making post-industrial sites a relevant setting for studying how learners interpret human–environment relations under instructional constraints (Yemini et al., 2025).

### 2.2 Experiential dimensions in narrative animation

Beyond presentation design, learning outcomes in multimedia environments are influenced by learners’ subjective experiences during viewing. As indicated by video-based learning research, experiential responses, including interest, engagement, emotional response, perceived comprehension, audiovisual experience, and perceived educational value, are closely linked to attention allocation, motivational regulation, and metacognitive monitoring (Fredricks et al., 2004). This study considers the above-mentioned responses as experiential dimensions of narrative animation, involving how learners appraise and regulate their interactions with the animation as it unfolds.

Interest is associated with sustained attention and deeper processing. Moreover, narrative formats can arouse situational interest through characters, plot structure, and visual style. Thus, learners are enabled to stress key ideas (Renninger and Hidi, 2016). Engagement is typically conceptualized as behavioral, emotional, and cognitive involvement in learning activities and is frequently employed as a proximal indicator of participation and attentional focus (Fredricks et al., 2004). The emotional response may influence attention allocation and subjective engagement, yet under brief and information-dense viewing conditions, it does not necessarily translate into knowledge gains. Moreover, under strong affective arousal, dramatic or salient details, rather than structurally important relationships, arouse greater attention (Loderer et al., 2020).

Perceived comprehension, with a close link to metacognitive monitoring processes, can reveal learners’ subjective judgments regarding their understanding. In brief multimedia learning episodes, however, perceived understanding can be inflated by fluency and enjoyment. Accordingly, discrepancies between subjective judgments and objective performance are formed (Loderer et al., 2020). The audiovisual experience and perceived educational value also play significant roles in video-based learning, particularly in evaluative responses. Favorable audiovisual appraisals may reflect processing fluency or focus on surface features, and therefore may not accurately indicate successful conceptual encoding when content density and time constraints are considerable (Noetel et al., 2022). Perceived educational value primarily reflects an evaluative appraisal and may be more directly relevant to value-oriented intentions than to objective knowledge performance, especially when learners form rapid judgments after a single brief viewing (Eccles and Wigfield, 2002). Reflective feedback captures learners’ evaluations of clarity, effectiveness, and potential improvements and may indicate an awareness of gaps in understanding rather than mastery itself (Shute, 2008).

Collectively, these experiential dimensions offer a multifaceted perspective for understanding learners’ responses to short narrative animations. However, prior research also indicates that under conditions of high conceptual density or time pressure, subjective experiential ratings and objective learning performance may not fully align (Plass et al., 2015). The potential for such misalignment to vary across developmental stages remains underexplored, particularly in the context of primary education. Within environmental education research, affective appraisals (e.g., hope, worry, and engagement) are often discussed as relevant to young people’s pro-environmental engagement, indicating that experiential responses can be consequential for care- and responsibility-oriented orientations even when understanding is still developing (Bird, 2024).

### 2.3 Cognitive and developmental factors in learning complex concepts

Children’s learning from multimedia materials is influenced by their cognitive development, metacognitive abilities, and socio-emotional maturation. Throughout the primary school years, improvements are observed in working memory capacity, the ability to identify main ideas, and effort regulation. These enhancements support more effective selection and integration of information from digital learning environments (Boetje et al., 2024). However, particularly in the lower primary grades, learners often encounter difficulties in planning, monitoring, and regulating their understanding when engaging with multimedia content. They may either follow narratives passively or focus predominantly on specific details unless strategic support is provided (Liu et al., 2022). These developmental factors suggest that differences between grade levels are more pronounced in evaluative interpretation and value-oriented responses than in immediate knowledge acquisition under time constraints.

A further issue is whether gains in CK lead to value-oriented intentions. In the fields of environmental and civic education, research shows that the correlation between knowledge and behavioral or support intentions is often modest, challenging linear “knowledge leads to behavior” models (Kollmuss and Agyeman, 2002). Recent environmental education research has likewise reported weak or non-significant relationships among environmental knowledge, pro-environmental beliefs, and pro-environmental behaviors among pre-service teachers, reinforcing the view that value-oriented outcomes often depend on norms, context, and motivational factors beyond knowledge acquisition alone (Obiagu et al., 2024). More broadly, affective responses, social norms, perceived efficacy, and contextual constraints are central to intention formation, indicating that knowledge constitutes only one component of value-oriented decision-making (Colombo et al., 2023). In heritage education, programs can affect students’ understanding of heritage and citizenship, and may also influence shifts in attitudes or self-reported participation intentions (Li et al., 2025). However, in the context of industrial heritage, which merges technical process understanding with historical meaning-making, it remains uncertain whether knowledge acquisition and value-oriented outcomes are systematically related, especially across different grade levels, under conditions of short narrative animation. In place-based environmental and sustainability education, “stewardship” is often treated as an early orientation toward care and responsibility for places rather than as direct behavioral change; for post-industrial landscapes, it therefore remains empirically open whether brief narrative encounters yield aligned gains in CK and stewardship-oriented intentions across grade levels.

Beyond the relationship between knowledge and intention, the conceptualization of stewardship also requires clarification. Bennett et al. (2018) conceptualize environmental stewardship as a sense of care, responsibility, and commitment towards sustaining valued places and environments rather than merely engaging in observable pro-environmental behaviors. Gruenewald (2003) further argues that place-based education develops learners’ relationships with local environments through meaningful engagement with place, thereby fostering place attachment and responsibility-oriented dispositions. Yemini et al. (2025) likewise suggest that place-based environmental and sustainability education encourages learners to interpret human–environment relationships through local contexts while cultivating sustainability-related responsibility alongside conceptual understanding. Drawing on these perspectives, Place-based Sustainability Stewardship Intention (PESI) is conceptualized in the present study as an early stewardship-oriented disposition reflecting children’s willingness to care for, value, and support the sustainable future of a specific place. Accordingly, PESI is distinguished from a general sustainability attitude, a discrete civic participation intention, or a purely evaluative judgement of the animation itself. Rather than representing direct behavioral change, PESI is treated as a place-specific value-oriented learning outcome that complements, rather than duplicates, content knowledge in animation-based environmental education.

### 2.4 Research gap and research questions

Previous studies have explored digital storytelling through various lenses, including multimedia learning, cognitive load, experiential responses, and developmental and value-oriented learning perspectives. Nonetheless, in the context of concept-dense and time-constrained narrative animations, classroom-based evidence remains limited. It is still unclear whether cognitive and value-oriented learning outcomes are influenced by similar or distinct factors and whether these outcomes develop in parallel or along different pathways. This limitation is especially evident in industrial heritage education, where technical complexity intersects with value-laden interpretation.

Accordingly, this study addresses the following three research questions:

*RQ1*: How does primary school students’ content knowledge (CK) of complex industrial heritage concepts change across baseline (T0), immediate post-viewing (T1), and two-week follow-up (T2) assessments of a brief digital storytelling animation?

*RQ2*: How are the seven experiential dimensions and grade level associated with CK immediately after viewing?

*RQ3*: How are CK and grade level associated with Place-based Sustainability Stewardship Intention (PESI)?

These research questions are addressed through complementary descriptive, regression, and structural equation modelling analyses. Specifically, RQ1 is examined using repeated knowledge assessments across T0, T1, and T2. RQ2 is examined through the CK component of the structural equation model. RQ3 is examined through the PESI component of the structural equation model, together with the supplementary regression analysis reported in Section 4.

### 2.5 Hypotheses

Grounded in multimedia learning theory, Cognitive Load Theory (CLT), and research on children’s cognitive development, this study distinguishes between two complementary outcome domains in brief, time-constrained narrative animation. CK reflects knowledge performance under processing constraints, whereas Place-based Sustainability Stewardship Intention (PESI) represents an early stewardship-oriented disposition that is expected to depend more strongly on developmental and experiential factors than on knowledge acquisition alone.

Accordingly, three theory-driven hypotheses corresponding to the research questions are proposed:

*H1* (Tests RQ2): Interest is positively associated with CK.

*H2* (Tests RQ3): Grade level is positively associated with PESI.

*H3* (Tests RQ3): CK is not expected to be strongly associated with PESI in brief narrative learning contexts.

Because existing theory does not provide sufficiently specific expectations for the relationships between the remaining experiential dimensions (engagement, emotional response, perceived comprehension, audiovisual experience, educational value, and reflective feedback) and the outcome variables, these associations are examined exploratorily under RQ2 rather than being formulated as confirmatory hypotheses.

## 3 Materials and methods

### 3.1 Research context and animation development

**Figure 1:**
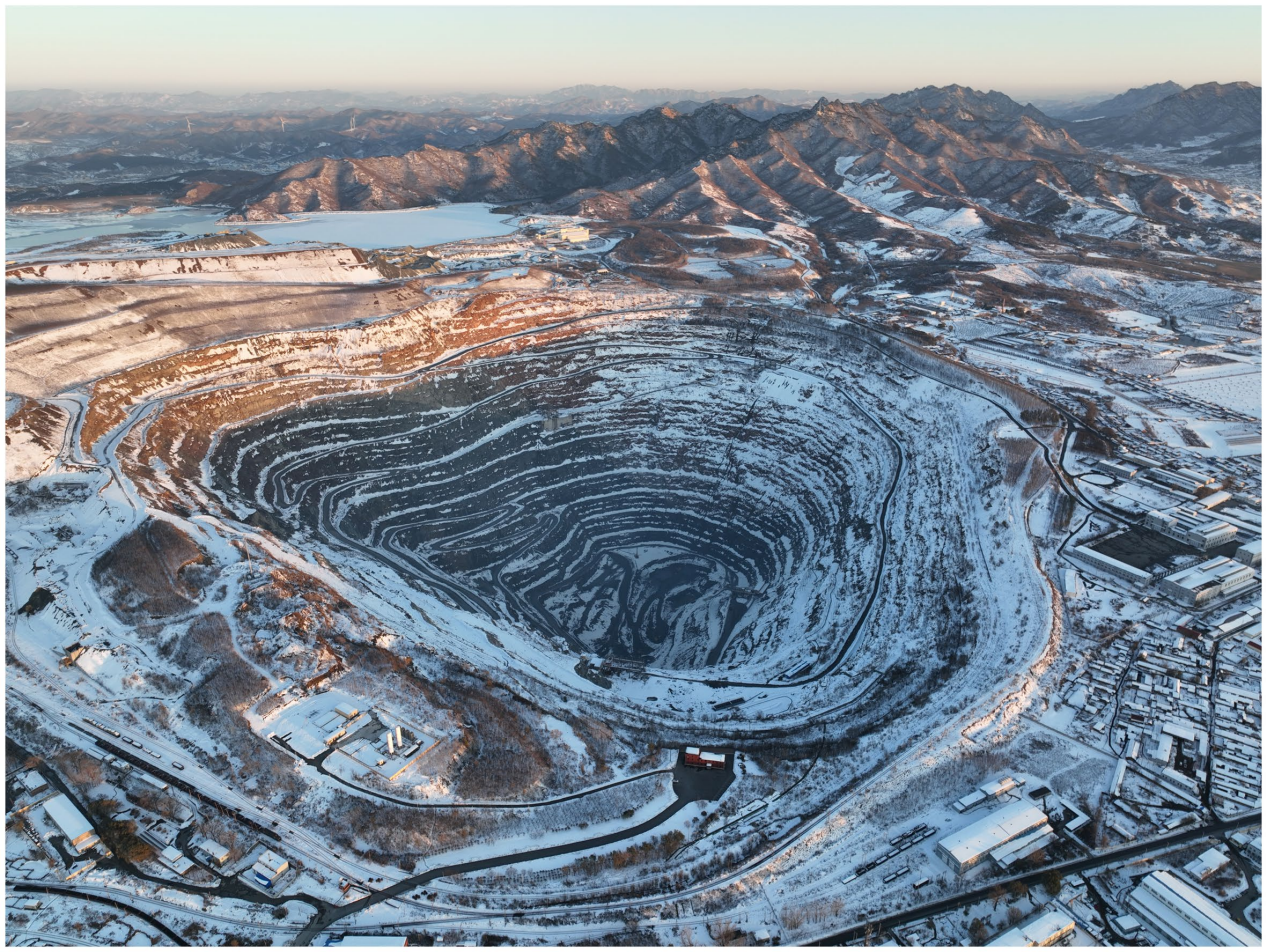
Aerial photograph taken in December 2023, showing the Dagushan Iron Mine after nearly a century of open-pit mining. Image source: the author.

Building on the preceding literature review and grounded in the historical and contemporary landscape context of the Dagushan Iron Mine (Figure 1), a post-industrial open-pit site shaped by nearly a century of extraction and currently undergoing ecological restoration, this study examines how primary school students engage with conceptually complex industrial heritage content through digital storytelling animation, and how these learning experiences relate to CK and PESI. Dagushan exemplifies a place-based environmental challenge in which industrial production, large-scale landscape transformation, and contemporary restoration imperatives intersect, making it a suitable context for examining how children interpret human–environment relations, environmental consequence, and the possibility of recovery within a short narrative format.

**Figure 2:**
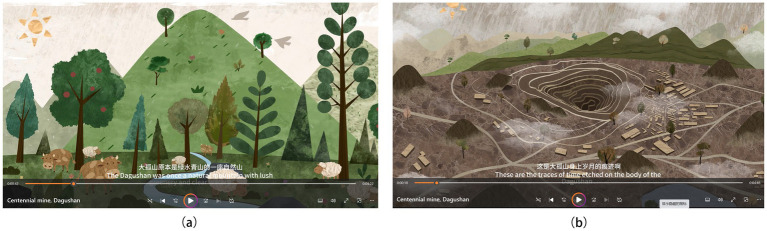
Scenes from the animation illustrating the Dagushan Iron Mine landscape before extraction **(a)** and after open-pit mining, showing the transition from a natural environment to an industrialized site **(b)**.

Importantly, the animation employed in this study was not an *ad hoc* instructional stimulus but the outcome of a systematically developed and empirically validated program of research conducted between 2022 and 2025 (Bian et al., 2025b; Bian et al., 2026). The animation presents a simplified narrative contrast between the pre-extraction landscape and the post-mining open-pit site (Figure 2), serving as the instructional stimulus for the classroom study. The final edited animation runs for 5 min 02 s and is organized into 20 discrete narrative shots, each combining a specific camera framing, on-screen action, and voiceover narration. The production storyboard specifies the temporal organization of these shots, with durations ranging from several seconds to approximately 30 s and longer segments concentrated around historically information-rich content (e.g., the transition from 1956 to the present and the spiral open-pit mining sequence). Together, the overall duration, shot structure, and temporal organization provide an objective description of the animation’s information density and pacing, thereby characterizing the processing constraints under which learners encoded concept-dense content during the classroom viewing episode. The animation was developed through an evidence-informed mixed-methods process integrating content appraisal, narrative structuring, and staged validation prior to classroom implementation. This developmental trajectory provides a traceable empirical foundation for the subsequent quantitative analyses and strengthens the interpretability of the findings. Detailed documentation of the development and validation procedures is provided in Supplementary material S1.

### 3.2 Participants and sampling

**Figure 3:**
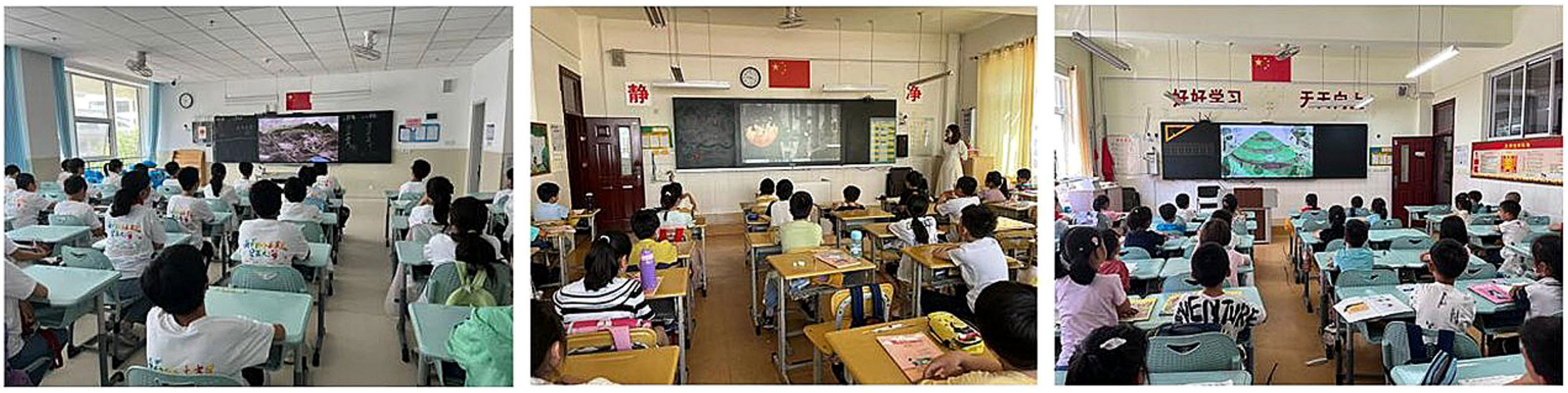
Scenes of students watching animations.

**Figure 4:**
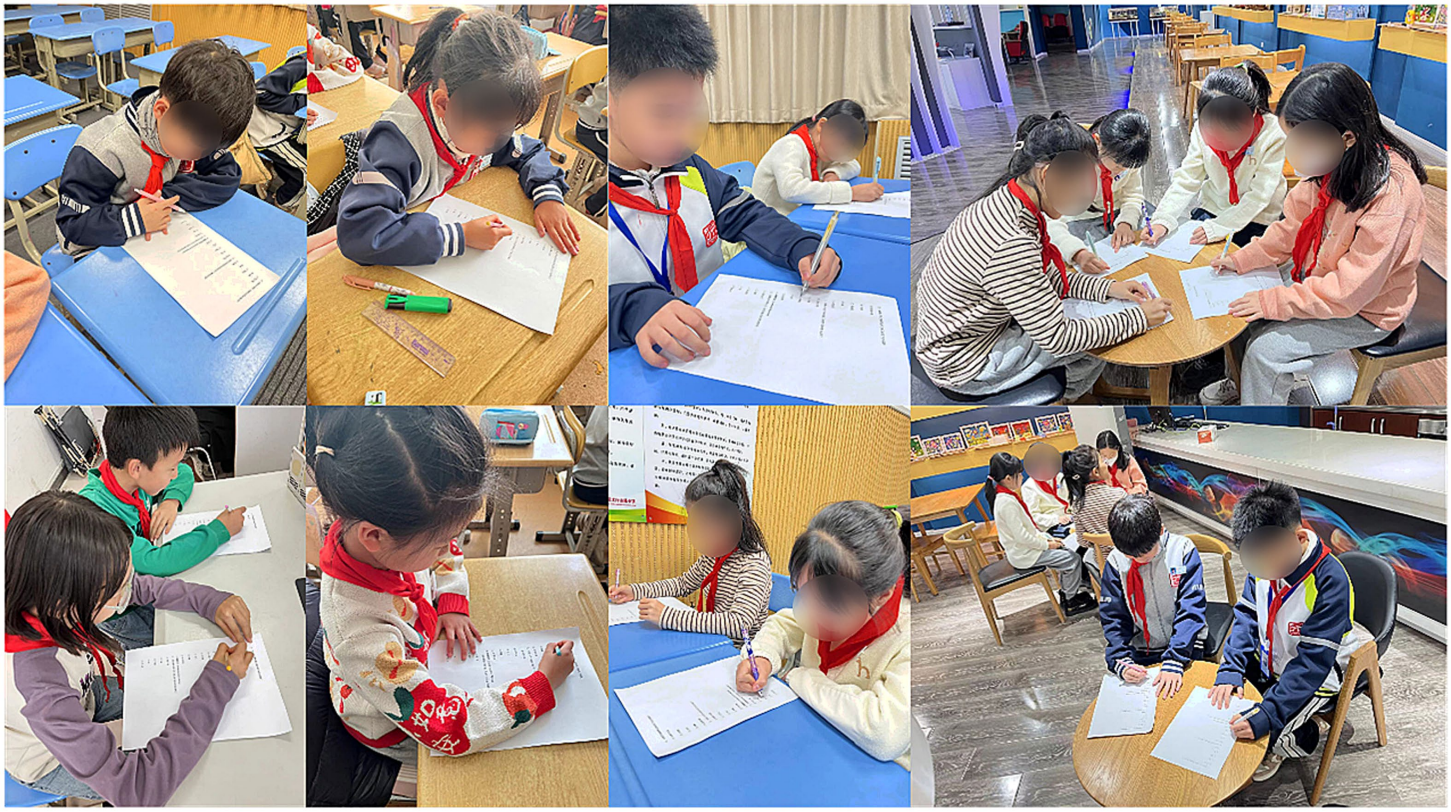
Participation of students of different grades in the survey questionnaire.

The target population consisted of primary school students from Years 1 to 6. With the assistance of the participating school and classroom teachers, individual students were randomly selected from class lists across Years 1–6 during regular school teaching. To ensure adequate representation at each grade level, participants were recruited from multiple classes within each grade. Data collection was coordinated with the school’s teaching schedule and conducted during normal classroom activities. A total of 470 questionnaires were collected. After excluding invalid cases (e.g., substantial missing sections or evidently patterned responses), 452 valid responses were retained, resulting in an effective response rate of 96.17%. The final sample included 72 students from Year 1 (15.93%), 68 from Year 2 (15.04%), 88 from Year 3 (19.47%), 68 from Year 4 (15.04%), 73 from Year 5 (16.15%), and 83 from Year 6 (18.36%). This distribution covered the lower, middle, and upper primary levels and enabled comparisons across grade levels. The classroom implementation of the animation viewing and the subsequent questionnaire administration are illustrated in Figures 3, 4.

All procedures adhered to institutional and national ethical guidelines for research involving children. Ethical approval had been secured prior to data collection. Written informed consent was obtained from the parents or legal guardians of participating students prior to data collection. Consent forms were distributed and collected through the participating school by the research team in February 2025, and assent was obtained from participating students before the classroom session. Participation was voluntary, and students could withdraw at any time without any penalty.

### 3.3 Data collection and instruments

Under standardized classroom conditions, students viewed the animation using an audio projector. Teachers supervised the session and followed a standardized administration protocol, providing no additional instructional explanations beyond the scripted instructions. To minimize the potential influence of reading ability on questionnaire responses, students in Years 1–3 completed a paper-based questionnaire with all items read aloud using a standardized script, whereas students in Years 4–6 completed an identical online questionnaire independently. This arrangement was adopted because younger primary school students have more limited reading fluency, and independent completion could introduce construct-irrelevant variance associated with reading comprehension rather than the target constructs being measured. Apart from the mode of questionnaire administration, all participants received identical questionnaire content, item order, response options, standardized instructions, classroom procedures, and animation viewing conditions to maximize measurement comparability across grade levels.

The research instrument comprised two components: (a) a Content Knowledge (CK) test and (b) a multidimensional perception scale. The CK test consisted of seven single-choice items designed to assess students’ understanding of the principal concepts presented in the animation. Collectively, the items covered historical development, landscape transformation, industrial production, mineral resources, spatial characteristics, and industrial heritage recognition. To ensure measurement consistency, the same CK test was administered at three time points: before viewing (T0), immediately after viewing (T1), and 2 weeks later (T2) during the supplementary cognitive-load session (see Section 4.6). This repeated-measures design enabled direct comparisons of students’ knowledge performance and concept acquisition over time using an identical instrument. A complete item blueprint, conceptual coverage, full item wording, correct-answer key, and item-level accuracy across T0, T1, and T2 are provided in Supplementary material S2.

The perception scale assessed students’ experiential responses to the animation across multiple learning-related dimensions. Detailed construct definitions and scale development procedures are reported in Section 3.4, and psychometric evidence regarding reliability and validity is presented in Section 3.5.

### 3.4 Perception scales

The second part of the questionnaire assessed students’ reactions to the animated content across experiential dimensions commonly examined in multimedia learning and educational psychology. Following established guidelines for measuring attitudes in children of similar ages (Mellor and Moore, 2014), all items were rated on a five-point Likert scale (1 = strongly disagree, 5 = strongly agree).

Items were adapted from validated instruments in multimedia learning, affective engagement, and perceived educational value, and were rephrased to fit the animation-viewing context and primary school students’ linguistic capabilities. The perception scale covered seven dimensions: Interest, Comprehension Ability, Emotional Response, Engagement, Audiovisual Experience, Educational Value, and Reflective Feedback. Standardized factor loadings for all perception-scale items are reported in Supplementary material S3.

To ensure content relevance, clarity, and conceptual coverage, all items were independently reviewed by three experts in educational technology and heritage education, and minor revisions were made based on their feedback. A pilot study with primary school students was then conducted to confirm age-appropriateness and response comprehension. When items were read aloud to younger students, teachers strictly adhered to a standardized script to minimize interpretive bias. Reliability and validity evidence for the final scales is reported in Section 3.5.

### 3.5 Reliability and validity testing

Instrument reliability and construct validity were examined using SPSS 26.0 and AMOS 23.0. Harman’s single factor test suggested limited common method bias (first factor = 23.49%, below the 40% threshold). Internal consistency for all perception constructs was acceptable (all Cronbach’s *α* > 0.75; overall α = 0.865). Construct validity was supported by EFA and CFA: the data were suitable for factor analysis (KMO = 0.845; Bartlett’s test χ^2^ = 5111.129, df = 465, *p* < 0.001), and the CFA measurement model showed strong fit (χ^2^/df = 1.033, RMSEA = 0.009, CFI = 0.997) with adequate convergent validity (all AVE > 0.50). The CK test comprised seven dichotomously scored items. Its measurement quality was established through expert review, content alignment, conceptual coverage, and item-level performance across repeated assessments. Detailed information, including the item blueprint, conceptual coverage, full item wording, correct-answer key, and item-level accuracy across T0, T1, and T2, is provided in Supplementary material S2. Additional psychometric outputs for the perception scales (e.g., full factor loadings and detailed reliability and validity results) are provided in Supplementary material S3. To further enhance research transparency, the anonymized participant-level datasets supporting these analyses have been deposited in the Zenodo repository^1^.

### 3.6 Data analysis

All quantitative analyses were conducted using SPSS version 26.0 and AMOS version 23.0. Initially, descriptive statistics were calculated for CK, PESI, and the experiential dimensions. Changes in Content Knowledge (CK) across the three assessment occasions (T0, T1, and T2) were analyzed using one-way repeated-measures analysis of variance (ANOVA) based on individually linked participant data. Where the omnibus test was significant, paired-samples *t*-tests with Cohen’s *d* were conducted as *post hoc* comparisons to examine changes between T0 and T1, T1 and T2, and T0 and T2. Pearson correlation analyses were then performed to examine the bivariate associations among the study variables. The experiential constructs demonstrated moderate positive intercorrelations (*r* = 0.289–0.630; see Supplementary material S4 for the complete correlation matrix), and the variance inflation factors (VIFs < 5) indicated no evidence of problematic multicollinearity.

Two analytical models were subsequently estimated to examine the remaining research questions. (a) As a preliminary, descriptive step, a multiple linear regression model predicted PESI from grade level and composite scores for each experiential dimension, computed by averaging the corresponding questionnaire items. This model is reported at the start of Section 4 as an initial descriptive overview and does not itself test the study hypotheses. (b) The structural equation model (SEM) that tests the hypotheses and addresses RQ2–RQ3 was specified using latent constructs for the seven experiential dimensions and PESI, each measured by three observed questionnaire items (see Section 4.4 and Supplementary material S3 for standardized factor loadings). Content Knowledge (CK) and grade level were included in the structural model as observed variables. Thus, composite scores were used only for the preliminary regression and were not used in the SEM estimation. All statistical tests were two-tailed with a significance level of *α* = 0.05. The explanatory power of the regression model was evaluated using *R*^2^ and adjusted *R*^2^, whereas SEM results were evaluated using global model fit indices and standardized path coefficients.

### 3.7 Supplementary follow-up cognitive load measurement

A supplementary cognitive load measurement was conducted 2 weeks after the primary data collection with the same cohort of students (*N* = 452) to examine retrospective perceived cognitive load as supplementary evidence for interpreting knowledge performance under time-constrained multimedia learning. Because this measurement was collected 2 weeks after viewing rather than during the animation itself, it captures students’ retrospective perceived cognitive load, that is, processing difficulty as recalled after a delay, rather than a concurrent, online measure of cognitive load during viewing; this distinction is revisited when interpreting the supplementary results (Section 5.2). Under the same classroom conditions as in the initial session, students completed a brief six-item cognitive load scale, which consisted of three extraneous-load items, two intrinsic-load items, and one mental-effort item. They then completed the same seven-item CK test used in the main study, along with two reverse-keyed items used as response validity checks. Instead of testing additional hypotheses, this supplementary measurement was intended to examine whether retrospective perceived cognitive load could provide additional supporting evidence for interpreting the associations between experiential perceptions and knowledge performance observed in the primary analyses.

## 4 Results

### 4.1 Changes in content knowledge across T0, T1, and T2

Changes in Content Knowledge (CK) across the three assessment occasions were examined using a one-way repeated-measures analysis of variance (ANOVA). A significant main effect of assessment occasion was observed, *F* (2, 902) = 975.36, *p* < 0.001, partial η^2^ = 0.684, indicating significant differences in CK performance across T0, T1, and T2. Mean CK scores increased from 1.71 (SD = 1.10) at baseline (T0) to 4.46 (SD = 1.23) immediately after viewing (T1), and further to 5.10 (SD = 1.31) at the two-week follow-up (T2).

Follow-up paired-samples *t*-tests indicated significant improvements between all assessment occasions. CK increased substantially from T0 to T1, *t* (451) = 34.47, *p* < 0.001, Cohen’s *d* = 1.62, followed by a further significant increase from T1 to T2, *t* (451) = 7.74, *p* < 0.001, Cohen’s *d* = 0.36. The cumulative improvement from T0 to T2 was also significant, *t* (451) = 41.61, *p* < 0.001, Cohen’s *d* = 1.96. Item-level accuracy rates across T0, T1, and T2 are presented in Supplementary material S2, providing item-level evidence for differential acquisition of individual concepts across the three assessment occasions. Complete statistical results are provided in Supplementary material S5.

### 4.2 Preliminary regression analysis

Before estimating the structural model, a preliminary multiple linear regression analysis was conducted to provide an initial overview of how experiential dimensions and grade levels relate to students’ PESI. The predictors included CK, Interest Level (I), Comprehension Ability (C), Emotional Response (ER), Educational Value (EV), Engagement (E), Audiovisual Experience (AE), Reflective Feedback (RF), and grade level. The regression model accounted for 42.4% of the variance in PESI (*R* = 0.651, *R*^2^ = 0.424). Several experiential variables, including Interest Level, Comprehension Ability, Emotional Response, Educational Value, Engagement, and Audiovisual Experience, demonstrated significant positive coefficients (all *ps* < 0.05). Grade level also showed a positive association with PESI for the upper-grade group (*p* < 0.001), whereas CK and Reflective Feedback were not significant predictors (*ps* > 0.20). Full descriptive statistics for the main study variables and the complete regression coefficient table are reported in Supplementary materials S6, S7, respectively.

### 4.3 Model fit

The structural model demonstrated acceptable overall fit to the observed data (χ^2^/df = 2.93; RMSEA = 0.065; CFI = 0.884; TLI = 0.862; NFI = 0.836). While χ^2^/df and RMSEA met conventional benchmarks, CFI, TLI, and NFI were below the commonly cited 0.90 criterion. Overall, the model demonstrated acceptable, although not optimal, fit to the observed data (Table 1).

**Table 1:** Model fit indices.

Fit index	Value
χ^2^/df	2.93
RMSEA	0.065
NFI	0.836
TLI	0.862
CFI	0.884

### 4.4 Measurement model

Before interpreting the structural paths, the measurement properties of the latent constructs were examined. For the seven latent constructs from the perception scale (Interest Level, Engagement, Emotional Response, Comprehension Ability, Audiovisual Experience, Educational Value, and Reflective Feedback), standardized factor loadings generally ranged from 0.66 to 0.84 and were statistically significant on their intended constructs (*p* < 0.001). Within the Interest construct, one indicator showed a comparatively lower loading (*λ* = 0.43) and was retained to preserve conceptual coverage of situational interest within the narrative learning context; retaining this item did not materially affect the construct-level reliability and validity indices (see Section 3.5). The three observed indicators of PESI exhibited factor loadings ranging from 0.85 to 0.89. In the model, CK served as an observed variable. Full factor loadings are reported in Supplementary material S3.

### 4.5 Structural model results

A structural equation model was estimated to examine associations among the experiential dimensions, CK, grade level, and PESI. Standardized path coefficients and significance tests are reported in Table 2. For CK, Interest showed the strongest standardized association with knowledge performance (*β* = 0.670), indicating that interest was the experiential factor most strongly associated with content knowledge in the structural model. Audiovisual Experience (*β* = −0.438, *p* < 0.001) and Educational Value (*β* = −0.198, *p* = 0.004) were negatively associated with CK. Engagement, Emotional Response, Comprehension Ability, Reflective Feedback, and grade level were not significantly associated with CK (all *ps* > 0.05). For PESI, grade level showed the strongest standardized association (*β* = 0.569), whereas CK was not significantly associated with PESI (*β* = −0.024, *p* = 0.557). Consistent with the non-significant CK → PESI path, no statistically significant indirect effects via CK were observed. These findings should be interpreted together with the repeated-measures analyses reported in Section 4.1, which demonstrated significant improvements in CK over time despite the absence of a direct association between CK and PESI.

**Table 2:** Standardized path coefficients of the structural equation model.

Path	*β*	*p*-value
Effects on CK		
Interest → CK	0.670ᵃ	—
Engagement → CK	−0.115	0.097
Emotional Response → CK	0.008	0.910
Comprehension → CK	−0.009	0.892
Audiovisual Experience → CK	−0.438	< 0.001
Educational Value → CK	−0.198	0.004
Reflective Feedback → CK	−0.011	0.867
Grade Level → CK	0.047	0.290
Effects on PESI		
CK → PESI	−0.024	0.557
Grade Level → PESI	0.569ᵃ	—

β = standardized path coefficient. 𝐿ixed reference parameter for model identification; not subject to a significance test.

### 4.6 Supplementary follow-up cognitive load results

A supplementary session was conducted 2 weeks after the main survey with the same cohort (*N* = 452) to examine retrospective perceived cognitive load and its associations with knowledge performance. Under the same classroom settings, students completed a brief cognitive-load scale and then completed the same seven-item CK test used in the main study. Extraneous cognitive load was negatively correlated with CK (*r* = −0.684, *p* < 0.001). Audiovisual Experience was positively correlated with extraneous load (*r* = 0.377, *p* < 0.001) and negatively correlated with CK (*r* = −0.264, *p* < 0.001). Educational Value was weakly positively correlated with CK (*r* = 0.132, *p* = 0.005) and negatively correlated with extraneous load (*r* = −0.186, *p* < 0.001). A regression model with CK as the dependent variable and extraneous load, Audiovisual Experience, and Educational Value as predictors accounted for 46.8% of the variance (*R*^2^ = 0.468, *F* = 132.45, *p* < 0.001). Extraneous cognitive load was the only statistically significant predictor (*B* = −1.693, *t* = −18.23, *p* < 0.001), whereas Audiovisual Experience (*B* = −0.022, *p* = 0.872) and Educational Value (*B* = 0.020, *p* = 0.914) were not significant after extraneous cognitive load was included. Neither Intrinsic Load (*r* = 0.090, *p* = 0.056) nor Mental Effort (*r* = 0.041) were significantly correlated with CK, suggesting that the observed pattern was specific to extraneous processing demands rather than reflecting general item difficulty or effort. In separate univariate regressions, Audiovisual Experience alone accounted for 7.0% of the variance in CK (*B* = −0.785, *p* < 0.001), but this association became non-significant once extraneous cognitive load was included in the multivariate model, consistent with a mediation-like pattern in which extraneous load carries the negative association between Audiovisual Experience and CK. Full descriptive statistics, the complete correlation matrix, and the univariate and multivariate regression tables for this supplementary follow-up are reported in Supplementary material S8. At the two-week follow-up (T2), the mean CK score reached 5.10 out of 7 (72.9% correct responses), indicating that students retained a substantial proportion of the knowledge acquired from the animation over the two-week interval.

## 5 Discussion

Although the learning context in this study is industrial heritage, the interpretation is intended to be transferable to environmental and sustainability education (ESE) settings where short-form videos or narrative animations are used to introduce concept-dense place-based issues. In particular, the findings speak to instructional situations in which brief media are tasked with communicating causal chains in human–environment relations, including environmental consequence and the possibility of restoration in post-industrial or degraded landscapes. Rather than treating short videos as uniformly effective or ineffective, the present analysis differentiates two outcome domains that can co-occur under tight pacing: (a) content knowledge (CK), reflecting the extent to which learners encode causal and conceptual information under processing constraints; and (b) PESI, reflecting learners’ evaluative orientations of care, responsibility, and willingness to engage with the place. Importantly, PESI is conceptualized in this study as an early, developmentally appropriate stewardship orientation rather than a behavioral commitment, capturing students’ emerging evaluative positioning toward place-based environmental care rather than enacted pro-environmental action. The results indicate that CK is best accounted for by interest-related attention and processing demand, whereas PESI is more strongly associated with grade level and learners’ immediate narrative appraisals. For ESE, this divergence highlights a practical risk in short-form media use: positive experience signals (e.g., feeling moved, finding the material meaningful, or rating it as high-quality) should not be treated as proxies for causal understanding. Instead, educators and researchers should explicitly assess whether learners have grasped key mechanisms and cause–effect relations, and provide grade-sensitive follow-up tasks (e.g., guided explanation, sequencing, or restoration-reasoning prompts) to support conceptual integration alongside stewardship-oriented engagement. From a critical ESE perspective, this is not an argument for ‘cold cognition’, but for a pedagogical stance that treats affect as a legitimate entry point while refusing to substitute emotional response for causal understanding and reflective stewardship reasoning.

### 5.1 Selective conceptual uptake in short narrative animation

A single five-minute viewing session was associated with substantial improvements in students’ Content Knowledge (CK), with mean scores increasing from 1.71 at baseline (T0) to 4.46 immediately after viewing (T1), and further to 5.10 at the two-week follow-up (T2). Repeated-measures analyses indicated significant improvements in CK from baseline to the immediate post-test, with further gains observed at the two-week follow-up, indicating that knowledge acquisition was both immediate and sustained over time. However, learning gains were not uniform across all concepts. For example, concepts related to historical events, landscape transformation, and mineral resources generally showed greater gains than concepts requiring institutional or temporal integration, such as industrial heritage recognition. Overall, concepts that were concrete, visually salient, and closely integrated into the narrative were acquired more readily than those requiring greater abstraction, temporal integration, or institutional understanding. This pattern suggests that short narrative animations facilitate learning in a selective manner, whereby concrete and sequential information is acquired more readily than abstract or integrative concepts. The finding is consistent with multimedia learning research showing that transient information effects and high conceptual density increase cognitive processing demands during brief instructional episodes (Plass et al., 2015). From an instructional perspective, these results indicate that short animations are effective for introducing complex heritage topics and establishing foundational understanding but are less effective in promoting deeper conceptual integration without additional instructional support. Item-level accuracy patterns reported in Supplementary material S2 further demonstrated that improvements varied across individual concepts, indicating that different types of knowledge were acquired at different rates. Although overall CK continued to improve modestly at the 2-week follow-up, the persistence of weaker performance on conceptually demanding items suggests that durable understanding depends not only on knowledge retention but also on opportunities for learners to organize, connect, and apply newly acquired knowledge. Accordingly, follow-up instructional activities, such as guided discussion, explanation tasks, or structured reflection, may be necessary to strengthen conceptual integration and support longer-term learning outcomes (Hamby and Jones, 2022).

### 5.2 Content knowledge under time constraints: interest and processing demand

Among the experiential dimensions examined, interest showed the strongest standardized association with CK when all experiential variables were included in the model simultaneously; because this path was fixed as the reference parameter for model identification, it was not subject to a significance test, but its magnitude (*β* = 0.670) was substantially larger than that of any other predictor. Among the remaining predictors, Audiovisual Experience and Educational Value were significantly and negatively associated with CK. This finding is consistent with research suggesting that situational interest is associated with sustained attention and deeper processing in learning contexts (Renninger and Hidi, 2016). It suggests that interest enhances the likelihood of learners allocating attention to the essential information required for accurate knowledge acquisition during short narrative viewing sessions. In contrast, engagement, emotional response, perceived comprehension, and reflective feedback did not emerge as significant predictors of CK in the multivariate model. This outcome aligns with evidence indicating that subjective experiences and perceived understanding do not necessarily correlate with objective performance in short, information-dense multimedia learning episodes (Loderer et al., 2020).

In the primary structural model, both Audiovisual Experience and Educational Value exhibited negative associations with CK. Supplementary analyses conducted 2 weeks later assessed retrospective perceived cognitive load rather than cognitive load during viewing and revealed that extraneous cognitive load was negatively related to CK and remained the sole significant predictor when extraneous load, Audiovisual Experience, and Educational Value were considered together. When extraneous cognitive load was included in the supplementary regression model, the associations of Audiovisual Experience and Educational Value with CK were no longer significant. These findings are broadly consistent with Cognitive Load Theory (CLT) and provide additional support for one possible processing-demand account of the observed pattern. Because cognitive load was measured retrospectively 2 weeks after viewing and the supplementary analyses were correlational, these findings should not be interpreted as direct evidence of processing mechanisms during viewing or as supporting causal inference. Therefore, the negative associations observed in the primary model are more appropriately interpreted as being consistent with a possible load-related pattern rather than as evidence that positive audiovisual or value appraisals directly impede learning.

Other explanations also remain plausible and cannot be ruled out with the present design: because Audiovisual Experience and Educational Value are moderately correlated with Interest and with each other (*r* = 0.289–0.630), their negative paths could partly reflect a suppressor-type effect once shared variance is partialled out, or construct overlap among appraisal items that share method variance. Alternatively, students who focused more on surface-level audiovisual features may have allocated less attention to structurally important content. These accounts are not mutually exclusive with a processing-demand interpretation, and distinguishing between them would require experimental manipulation of presentation features. Experimental studies that manipulate pacing, signaling, or visual density are needed to more directly examine this interpretation.

### 5.3 Experiential correlates of value-oriented intentions

Contrary to the patterns observed with CK, PESI was associated with a broader array of experiential responses. In the regression analyses, interest, perceived comprehension, emotional response, engagement, audiovisual experience, and educational value all exhibited positive associations with support-oriented intentions, even when controlling for grade level. This finding is consistent with research suggesting that affective engagement, perceived relevance, and evaluative appraisals are significant contributors to intention-related outcomes in narrative and heritage learning contexts (Steriopoulos et al., 2024). Unlike performance-based outcomes, which were selectively predicted, support-oriented intentions seem to be more closely linked to learners’ appraisals of the narrative experience as meaningful, engaging, and valuable. More broadly, early childhood ESD research shows that value-oriented orientations and sustainability-linked action tendencies can be fostered through pedagogical experiences that prompt learners to interpret local environmental issues, even in intervention formats very different from short narrative viewing, suggesting that such orientations need not depend on full cognitive mastery (Kalafati et al., 2025). Taken together, these results indicate that support-oriented intentions are more closely explained by learners’ immediate narrative appraisals than by the performance-linked accounts that best describe CK. In practice, favorable experience ratings and higher PESI can rise alongside uneven conceptual uptake, and knowledge gains do not necessarily translate into support intentions within a single brief intervention.

### 5.4 Developmental sensitivity of value-oriented outcomes

The association of grade level with PESI was positive, whereas its correlation with CK was not statistically significant once experiential variables were controlled. This pattern indicates that grade-related differences are more apparent in evaluative responses than in immediate knowledge acquisition under time constraints. Developmental research shows that evaluative judgment, perspective-taking, and the capacity to contextualize narratives within broader social and historical frameworks develop throughout the primary school years (Case, 1992). One interpretation is that older students may be better equipped to interpret the animation in the context of wider historical or civic meanings, thereby forming stronger support intentions, even when knowledge test performance does not systematically vary by grade. Conversely, younger students may react strongly to narrative and perceptual features, yet these responses may be less tied to stable evaluative commitments. This interpretation remains tentative and warrants further investigation using age-sensitive measures of reasoning, value appraisal, and reflective judgment (Eisazadeh and Rajendram, 2020).

### 5.5 The limited association between content knowledge and support intentions

Content knowledge did not exhibit a significant association with PESI in the structural model. This result is consistent with critiques of the assumption that linear knowledge acquisition leads to behavioral changes in environmental and heritage education. These critiques suggest that knowledge alone is often insufficient for predicting behavioral or support-oriented intentions (Udall et al., 2021). This pattern echoes recent findings in environmental education showing that knowledge may not translate into pro-environmental beliefs or behaviors in a straightforward way, even when an educational intervention is present (Obiagu et al., 2024). In this study, support intentions were more strongly correlated with learners’ narrative appraisals, such as engagement, emotional response, and perceived relevance, than with the immediate mastery of factual or integrative knowledge. Accordingly, CK and PESI should be treated as related but non-equivalent outcomes that may co-occur within the same lesson without forming a single linear pathway.

## 6 Implications

On the theoretical level, this study contributes to a more nuanced understanding of learning from brief, concept-dense digital storytelling animations. The findings challenge the notion of a singular learning mechanism by suggesting that cognitive acquisition and value-oriented intentions are two parallel, yet non-equivalent, outcome domains under time constraints. CK is most consistent with a processing-demand account, in which interest facilitates essential processing and retrospective perceived extraneous cognitive load is associated with reduced conceptual integration. This is consistent with principles of multimedia learning and CLT (Sweller et al., 2019). Conversely, PESI is more closely related to developmental levels and learners’ narrative appraisals, echoing broader critiques of the simple “knowledge leads to behavior” assumptions in environmental and heritage education (Kollmuss and Agyeman, 2002). This distinction helps explain why brief narrative animations can elicit strong positive impressions and intentions even when conceptual uptake remains selective.

From a methodological perspective, the results underscore the importance of distinguishing between cognitive and value-oriented outcomes in both measurement and analysis. Relying on experiential ratings or attitudinal responses as proxies for learning can mask significant differences between subjective experience and objective performance in short multimedia interventions, as evidenced by previous research on perceived versus actual understanding (Loderer et al., 2020). The method of combining repeated assessments of content knowledge with a delayed follow-up assessment of retrospective perceived cognitive load offers a valuable approach for interpreting uneven learning patterns and for identifying instances where favorable experience ratings may reflect processing fluency rather than durable understanding. Additionally, the findings recommend explicitly modeling grade level when examining value-oriented outcomes, as developmental differences might be more pronounced in evaluative responses than in immediate knowledge scores once experiential variables are controlled.

## 7 Limitations and future directions

Although this study offers classroom-based evidence on how primary school students react to a short, concept-dense digital storytelling animation about industrial heritage, several limitations must be acknowledged and addressed in future research.

First, the instructional intervention was based on a brief digital storytelling animation, delivered within the strict time constraints typical of classroom pacing. Although this format reflects authentic instructional practice and the ecology of short-form media use, it limits the extent of conceptual unpacking and integrative meaning-making possible during the viewing. The limited duration may favor the uptake of concrete and sequential information, while potentially leaving institutionally framed, abstract, or integrative concepts more susceptible to incomplete processing. Future studies should explore alternative temporal structures, such as extended versions, segmented delivery, or spaced re-exposure designs, to determine whether additional processing time selectively enhances integrative understanding without reducing value-oriented engagement.

Second, the study was conducted under natural classroom conditions, enhancing ecological validity but reducing experimental control over classroom-related influences. Although standardized procedures were implemented throughout data collection, classroom context and differences in questionnaire administration across age groups cannot be completely separated from developmental differences. Consequently, the observed grade-level associations, particularly those involving PESI, cannot be attributed exclusively to developmental differences. In addition, because participants were drawn from multiple classrooms within the same school, classroom-level factors, such as differences in teacher practices, peer interactions, and classroom climate, may also have influenced students’ responses. Future research should incorporate classroom-level contextual measures and adopt analytical approaches that better account for hierarchical educational data.

Third, a supplementary cognitive-load session conducted 2 weeks after the main survey provides correlational support for interpreting the negative paths observed in the primary structural model as load-related, but it does not establish causality. Participants re-engaged with the animation content and completed both a cognitive-load scale and the same content-knowledge test, allowing for an examination of the distribution of extraneous load in relation to performance. However, since extraneous load was not experimentally manipulated, the analysis cannot confirm whether higher load directly caused lower knowledge performance, or if other unmeasured factors might have concurrently influenced both variables. Future studies should more directly test the proposed mechanism through experimental manipulations of extraneous processing demands, such as systematically varying signaling, pacing, redundancy, or visual complexity while keeping the content constant, and should also distinguish among alternative explanations for the negative associations observed in the structural model, including suppressor effects, construct overlap among experiential dimensions, and learners’ attention to surface audiovisual features.

Fourth, although the study distinguishes between cognitive outcomes and value-oriented outcomes, both domains were primarily assessed using self-report measures (for experiential dimensions and PESI) and a brief dichotomous content-knowledge test. While self-report instruments are effective for capturing learners’ appraisals in authentic settings, they are prone to fluency-based judgments and may not accurately reflect objective performance in brief multimedia episodes. Additionally, a seven-item knowledge test limits content coverage and may not adequately capture deeper conceptual integration. Future research could enhance measurement accuracy by expanding the knowledge assessment to include items that target integrative reasoning, transfer, or explanation-based understanding, and by correlating self-reported experiences with process measures such as response-time indicators, brief comprehension checks embedded during viewing, or structured recall and explanation tasks.

Fifth, the study was conducted within a single school context and focused on a single industrial heritage site, which limits the generalizability of the findings across different heritage types, instructional settings, and cultural interpretations of industrial heritage. The content related to industrial heritage is value-laden and context-dependent, and students’ value appraisals may vary when heritage is framed through perspectives such as local identity, national history, environmental restoration, or technological achievement. Future studies should investigate whether the parallel-outcome pattern observed in this study is replicable across various industrial heritage cases and diverse educational contexts, including museum-based programs and schools with different levels of exposure to heritage-related topics.

Finally, the proposed parallel-outcome framework was developed and examined within the context of a single animation-based industrial heritage program. Although the findings provide initial empirical support for this framework, it should be regarded as a preliminary explanatory framework that requires further validation across different educational settings and learner populations. Future studies should compare alternative theoretical models and evaluate the proposed framework using independent samples and diverse educational contexts.

## Data Availability

The datasets presented in this study can be found in online repositories. The names of the repository/repositories and accession number(s) can be found in the article/Supplementary material.

## Footnotes

1
https://doi.org/10.5281/zenodo.21212642

## Glossary

Content Knowledge (CK): Students’ objective performance on the knowledge test of animation content
Cognitive load: Mental processing demand placed on working memory during learning.
Complex concept learning: Learning requires integrating multiple ideas, causal relations, and abstractions
Extraneous cognitive load: Processing demand caused by presentation that does not directly support learning
Experiential dimensions: Learners’ immediate appraisals (e.g., interest, emotion, engagement) during viewing
Place-based Sustainability Stewardship Intention (PESI): Students’ intention to care for, visit, or protect industrial heritage

## References

[ref1] Bennett N. J. Whitty T. S. Finkbeiner E. M. Pittman J. Bassett H. Gelcich S. . (2018). Environmental stewardship: a conceptual review and analytical framework. Environ. Manag. 61, 597–614. doi: 10.1007/s00267-017-0993-2, 29387947 PMC5849669

[ref2] Bian X. Brown A. Marques B. (2025a). Examining a primary education approach using digital storytelling: Chinese industrial heritage as a vehicle to support learning. Heritage 8:477. doi: 10.3390/heritage8110477

[ref3] Bian X. Brown A. Marques B. (2025b). Heritage appreciation and awareness: a child educational approach exploiting animated video. Int. J. Art Des. Educ. 44, 286–304. doi: 10.1111/jade.12563

[ref4] Bian X. Brown A. Marques B. (2026). Balancing ecological restoration and industrial landscape heritage values through a digital narrative approach: a case study of the Dagushan iron mine, China. Land 15:155. doi: 10.3390/land15010155

[ref5] Bianchi G. Pisiotis U. Cabrera Giraldez M. (2022) in GreenComp – The European Sustainability Competence Framework, eds. Punie Y. Bacigalupo M. (Luxembourg: Publications Office of the European Union).

[ref6] Bird Á. (2024) Exploring Community Stewardship through place-based Learning: A case Study from the Burren. Doctoral dissertation, University of Galway, Galway, Ireland

[ref7] Boetje J. van Ginkel S. O. Smakman M. H. J. Barendsen E. Versendaal J. (2024). Information problem solving during a digital authentic task: a thematic analysis of students' strategies. Comput. Hum. Behav. Rep. 15:100470. doi: 10.1016/j.chbr.2024.100470

[ref8] Case R. (1992). The Mind's Staircase: Exploring the Conceptual Underpinnings of Children's Thought and Knowledge. Hillsdale, NJ: Lawrence Erlbaum Associates.

[ref9] Castro-Alonso J. C. Ayres P. Sweller J. (2019). “Instructional visualizations, cognitive load theory, and visuospatial processing,” in Visuospatial Processing for Education in Health and natural Sciences, (Cham: Springer International Publishing), 111–143.

[ref10] Cocal-Smith V. Hinchliffe G. Petterson M. G. (2023). Digital tools for the promotion of geological and mining heritage: case study from the Thames goldfield, Aotearoa, New Zealand. Geosciences 13:253. doi: 10.3390/geosciences13080253

[ref11] Colombo S. L. Chiarella S. G. Raffone A. Simione L. (2023). Understanding the environmental attitude-behaviour gap: the moderating role of dispositional mindfulness. Sustainability 15:7285. doi: 10.3390/su15097285

[ref12] de Koning B. Tabbers H. K. Rikers R. M. J. P. Paas F. (2007). Attention cueing as a means to enhance learning from an animation. Appl. Cogn. Psychol. 21, 731–746. doi: 10.1002/acp.1346

[ref13] Eccles J. S. Wigfield A. (2002). Changes in children’s self-competence and values: gender and domain differences across grades one through twelve. Child Dev. 73, 509–527. doi: 10.1111/1467-8624.00421, 11949906

[ref14] Eisazadeh N. Rajendram S. (2020). Supporting young learners through a multimodal digital storytelling activity. J. Early Child. Educ. Res. 9, 76–98.

[ref15] Fadhil A. Al-Alwan H. A. S. (2026). Visual storytelling and place-based learning: a generative approach to architectural cultural awareness. J. Asian Archit. Build. Eng., 1–17. doi: 10.1080/13467581.2026.2641354

[ref16] Fredricks J. A. Blumenfeld P. C. Paris A. H. (2004). School engagement: potential of the concept, state of the evidence. Rev. Educ. Res. 74, 59–109. doi: 10.3102/00346543074001059

[ref17] Gruenewald D. A. (2003). The best of both worlds: a critical pedagogy of place. Educ. Res. 32, 3–12. doi: 10.3102/0013189X032004003

[ref18] Hamby A. Jones N. (2022). The effect of affect: an appraisal theory perspective on emotional engagement in narrative persuasion. J. Advert. 51, 116–131. doi: 10.1080/00913367.2021.1981498

[ref19] Hernandez Gonzalez F. (2023). Exploring the affordances of place-based education for advancing sustainability education: the role of cognitive, socio-emotional and behavioural learning. Educ. Sci. 13:676. doi: 10.3390/educsci13070676

[ref20] Hill D. Ameenuddin N. Reid Chassiakos Y. L. Cross C. Hutchinson J. Levine A. . (2016). Media and young minds. Pediatrics 138:e20162591. doi: 10.1542/peds.2016-2591, 27940793

[ref21] Kalafati M. Flogaiti E. Daskolia M. (2025). Enhancing preschoolers’ creativity through art-based environmental education for sustainability. Environ. Educ. Res. 31, 46–73. doi: 10.1080/13504622.2023.2291319

[ref22] Kalyuga S. (2011). Cognitive load theory: how many types of load does it really need? Educ. Psychol. Rev. 23, 1–19. doi: 10.1007/s10648-010-9150-7

[ref23] Kollmuss A. Agyeman J. (2002). Mind the gap: why do people act environmentally and what are the barriers to pro-environmental behavior? Environ. Educ. Res. 8, 239–260. doi: 10.1080/13504620220145401

[ref24] Li R. Song Q. Wang Y. Sun B. (2025). Perceived value and behavioral intentions of cultural heritage visitors: a SEM analysis using Lingnan classical gardens as a case study. Buildings 15:4070. doi: 10.3390/buildings15224070

[ref25] Liu R. Xu X. Yang H. Li Z. Huang G. (2022). Impacts of cues on learning and attention in immersive 360-degree video: an eye-tracking study. Front. Psychol. 12:792069. doi: 10.3389/fpsyg.2021.792069, 35153916 PMC8828640

[ref26] Loderer K. Pekrun R. Lester J. C. (2020). Beyond cold technology: a systematic review and meta-analysis on emotions in technology-based learning environments. Learn. Instr. 70:101162. doi: 10.1016/j.learninstruc.2018.08.002

[ref27] Makransky G. Petersen G. B. (2021). The cognitive affective model of immersive learning (CAMIL): a theoretical research-based model of learning in immersive virtual reality. Educ. Psychol. Rev. 33, 937–958. doi: 10.1007/s10648-020-09586-2

[ref28] Mayer R. E. (2024). The past, present, and future of the cognitive theory of multimedia learning. Educ. Psychol. Rev. 36:8. doi: 10.1007/s10648-023-09842-1

[ref29] Mellor D. Moore K. A. (2014). The use of Likert scales with children. J. Pediatr. Psychol. 39, 369–379. doi: 10.1093/jpepsy/jst079, 24163438

[ref30] Modi S. Gupta T. Rahmatullah M. (2024). Digital storytelling as a tool for global citizenship and sustainability: enhancing cross-cultural understanding in education. J. Interdiscip. Stud. Educ. 13. doi: 10.32674/s817ax14

[ref31] Noetel M. Griffith S. Delaney O. Harris N. R. Sanders T. Parker P. . (2022). Multimedia design for learning: an overview of reviews with meta-meta-analysis. Rev. Educ. Res. 92, 413–454. doi: 10.3102/00346543211052329

[ref32] Obiagu A. Ocheje J. Ofodum I. Eze E. (2024). Fostering environmental personal norms: the role of environmental education in Nigerian pre-service teachers’ environmental knowledge, pro-environmental beliefs and behaviours. Environ. Educ. Res. 30, 1231–1246. doi: 10.1080/13504622.2023.2297159

[ref33] Otto T. (2025). Should educators be concerned? The impact of short videos on rational thinking and learning: a comparative analysis. Comput. Educ. 234:105330. doi: 10.1016/j.compedu.2025.105330

[ref34] Peretz R. (2025). Integrating systems thinking into sustainability education: an overview with educator-focused guidance. Educ. Sci. 15:1685. doi: 10.3390/educsci15121685

[ref35] Plass J. L. Homer B. D. Kinzer C. K. (2015). Foundations of game-based learning. Educ. Psychol. 50, 258–283. doi: 10.1080/00461520.2015.1122533

[ref36] Renninger K. A. Hidi S. (2016). The power of Interest for Motivation and Engagement. New York, NY: Routledge.

[ref37] Shute V. J. (2008). Focus on formative feedback. Rev. Educ. Res. 78, 153–189. doi: 10.3102/0034654307313795

[ref38] Soysal N. Wilder R. Copsey O. Milligan L. O. (2026). Conceptualising a justice approach to environmental education in the global south: six pedagogical dimensions. Environ. Educ. Res. 32, 964–983. doi: 10.1080/13504622.2025.2545537

[ref39] Steriopoulos E. Khoo C. Wong H. Y. Hall J. Steel M. (2024). Heritage tourism brand experiences: the influence of emotions and emotional engagement. J. Vacat. Mark. 30, 489–504. doi: 10.1177/13567667231152930

[ref40] Sweller J. (2020). Cognitive load theory and educational technology. Educ. Technol. Res. Dev. 68, 1–16. doi: 10.1007/s11423-019-09701-3

[ref41] Sweller J. van Merriënboer J. J. G. Paas F. (2019). Cognitive architecture and instructional design: 20 years later. Educ. Psychol. Rev. 31, 261–292. doi: 10.1007/s10648-019-09465-5

[ref42] Tzima S. Styliaras G. Bassounas A. Tzima M. (2020). Harnessing the potential of storytelling and mobile technology in intangible cultural heritage: a case study in early childhood education in sustainability. Sustainability 12:9416. doi: 10.3390/su12229416

[ref43] Udall A. M. de Groot J. I. M. De Jong S. B. Shankar A. (2021). How I see me: a meta-analysis investigating the association between identities and pro-environmental behaviour. Front. Psychol. 12:582421. doi: 10.3389/fpsyg.2021.582421, 33796041 PMC8008126

[ref44] UNESCO Education 2030: Incheon declaration and framework for action for the implementation of sustainable development goal 4 (2016) Available online at: https://unesdoc.unesco.org/ark:/48223/pf0000245656

[ref45] Viteri F. Clarebout G. Crauwels M. (2013). Children’s recall and motivation for an environmental education video with supporting pedagogical materials. Environ. Educ. Res. 19, 228–247. doi: 10.1080/13504622.2013.771734

[ref46] Yemini M. Engel L. Ben Simon A. (2025). Place-based education–a systematic review of literature. Educ. Rev. 77, 640–660. doi: 10.1080/00131911.2023.2177260

