# Supplementary material for: How children learn complex environmental concepts through digital storytelling: divergent pathways to knowledge and stewardship intention
Source: Front Psychol. 2026 Jul 31;17:1893526. doi: 10.3389/fpsyg.2026.1893526 (PMC13473992; doi:10.3389/fpsyg.2026.1893526)
Supplement: Supplementary file 1 [file Supplementary_file_1.DOCX]

**Supplementary S1. Overview of the mixed-methods research process (2022–2025)**

This supplement documents the evidence-informed development and validation trajectory of the digital storytelling animation prior to classroom implementation. It is provided to demonstrate that the instructional stimulus used in the quantitative study was not ad hoc, but the outcome of a systematically staged research process.

To ensure that the digital storytelling animation was theoretically grounded and pedagogically appropriate for classroom use prior to quantitative evaluation, a staged mixed-methods research process was conducted between 2022 and 2025. This process comprised three interrelated phases: (1) research grounding and needs identification, (2) instructional development and narrative construction, and (3) pre-implementation validation.

**Phase 1:** Research grounding and needs assessment.

An integrative literature review and preliminary school-based investigations were undertaken to identify gaps in existing research and classroom practice. This scoping process indicated that empirical research on digital storytelling for industrial heritage education at the primary school level remains limited, with most prior studies focusing on museum contexts or high-immersion technologies. Teachers reported a lack of age-appropriate instructional resources for industrial heritage topics, while students consistently expressed a preference for animation-based learning formats. This phase established both the research rationale and the selection of animation as the instructional medium.

**Phase 2:** Case selection and instructional development.

The Dagushan Iron Mine was selected as the focal case through multiple rounds of field investigation and historical documentation. On-site observation, archival materials, and oral-history sources were triangulated to establish the narrative foundation. An expert-based value appraisal process was then conducted to prioritise key heritage elements and educational messages. Based on these inputs, a five-minute digital storytelling animation titled *Centennial Mine: Dagushan* was produced, employing an intergenerational family narrative to convey long-term industrial development and environmental transformation in a form aligned with primary students’ cognitive characteristics.

**Phase 3:** Staged validation prior to classroom implementation.

Before large-scale classroom use, the animation underwent expert review and small-scale student testing to evaluate historical accuracy, narrative coherence, scene clarity, and age-appropriateness. Targeted revisions were made where necessary. Following this validation, the classroom-based quantitative study reported in the main paper was conducted with students from Years 1–6 (N = 452).

By integrating evidence-informed content development, stakeholder input, and staged validation within a unified research framework, the study minimised the likelihood that observed classroom outcomes were artefacts of material quality or ad hoc design choices.

**Supplementary S2. Content Knowledge (CK) item blueprint, conceptual coverage, full item wording, correct answers, and item-level accuracy across T0, T1, and T2**

This supplement provides the item blueprint, conceptual coverage, full item wording, correct-answer key, and item-level accuracy for the seven-item Content Knowledge (CK) test across baseline (T0), immediate post-viewing (T1), and two-week follow-up (T2), providing additional evidence for the content validity of the CK instrument and the interpretation of selective concept acquisition across repeated assessments. The CK test was designed as a brief achievement test assessing students' understanding of the principal concepts presented in the animation rather than as a reflective psychometric scale. Each item measured a distinct concept related to historical development, industrial production, spatial characteristics, environmental transformation, or industrial heritage recognition. Collectively, the seven items covered the major historical, industrial, spatial, environmental, and heritage-management concepts presented in the animation. Measurement quality was established through expert review, content alignment, conceptual coverage, and item-level performance across repeated assessments rather than high internal consistency.

**Table S1. Content Knowledge (CK) item blueprint, wording, answer key, and item-level accuracy across T0, T1, and T2**

| Item | Concept assessed | Full item wording | Correct answer | T0 (%) | T1 (%) | T2 (%) |
| --- | --- | --- | --- | --- | --- | --- |
| CK1 | Historical landscape before mining | What was Dagushan like in its early historical development? | A beautiful natural mountain with a pleasant environment. | 35.8 | 81.6 | 73.0 |
| CK2 | Japanese occupation timeline | When did Japan invade Dagushan? | 1916 | 14.2 | 67.3 | 76.5 |
| CK3 | Post-war historical transition | When did the Soviet Union invade Dagushan? | 1949 | 17.3 | 66.4 | 71.2 |
| CK4 | Industrial development milestone | When did Dagushan change the history of Ansteel from having only iron to producing steel? | 1956 | 18.8 | 55.3 | 72.8 |
| CK5 | Mineral resource type | What rich mineral resources do Dagushan have? | Iron mine | 31.9 | 70.4 | 73.7 |
| CK6 | Mine depth and spatial scale | How deep has the Dagushan Iron Mine pit been excavated? | −414 m | 40.0 | 70.8 | 73.0 |
| CK7 | Industrial heritage recognition | When was Dagushan listed as an industrial heritage site in Liaoning Province? | 2017 | 12.8 | 33.8 | 69.9 |

Note: Percentages represent the proportion of students answering each item correctly (item difficulty; *p*-value) at baseline (T0), immediate post-viewing (T1), and the two-week follow-up (T2). For dichotomously scored knowledge tests, the proportion of correct responses is conventionally reported as the item difficulty (proportion correct), with higher values indicating easier items. The observed changes across the three assessment occasions provide item-level evidence supporting the interpretation of selective concept acquisition discussed in the main text. Statistical comparisons of CK performance across T0, T1, and T2 are reported in Supplementary S5.

**Supplementary S3. Measurement model factor loadings**

This supplement provides full transparency for SEM measurement properties.

**Table S2.Measurement model factor loadings**

| Latent Construct | Indicator | Standardised Loading |
| --- | --- | --- |
| Interest Level | I1 | 0.720 |
|  | I2 | 0.738 |
|  | I3 | 0.430 |
| Engagement | E1 | 0.793 |
|  | E2 | 0.776 |
|  | E3 | 0.704 |
| Emotional Response | ER1 | 0.843 |
|  | ER2 | 0.816 |
|  | ER3 | 0.732 |
| Comprehension Ability | C1 | 0.834 |
|  | C2 | 0.824 |
|  | C3 | 0.794 |
| Audiovisual Experience | AE1 | 0.726 |
|  | AE2 | 0.660 |
|  | AE3 | 0.711 |
| Educational Value | EV1 | 0.677 |
|  | EV2 | 0.701 |
|  | EV3 | 0.739 |
| Reflective Feedback | RF1 | 0.788 |
|  | RF2 | 0.763 |
|  | RF3 | 0.753 |
| PESI | PESI1 | 0.874 |
|  | PESI2 | 0.855 |
|  | PESI3 | 0.887 |

Note. All factor loadings were statistically significant (p < 0.001). One interest indicator (I3) showed a comparatively lower loading (λ = 0.43) but was retained to preserve conceptual coverage of situational interest.

**Supplementary S4. Correlations among the exogenous latent constructs in the structural model**

This supplement provides the complete correlation matrix among the seven exogenous latent constructs (Interest, Engagement, Emotional Response, Comprehension Ability, Audiovisual Experience, Educational Value, and Reflective Feedback), which were allowed to correlate freely in the structural model. It supports the summary statement in Section 3.6 that these constructs demonstrated moderate positive intercorrelations (r = 0.289-0.630).

**Table S3. Correlations among the exogenous latent constructs**

| Construct | I | E | ER | C | AE | EV | RF |
| --- | --- | --- | --- | --- | --- | --- | --- |
| Interest (I) | 1.000 | 0.489*** | 0.517*** | 0.419*** | 0.630*** | 0.482*** | 0.349*** |
| Engagement (E) | - | 1.000 | 0.489*** | 0.380*** | 0.353*** | 0.343*** | 0.358*** |
| Emotional Response (ER) | - | - | 1.000 | 0.385*** | 0.289*** | 0.402*** | 0.477*** |
| Comprehension Ability (C) | - | - | - | 1.000 | 0.406*** | 0.319*** | 0.313*** |
| Audiovisual Experience (AE) | - | - | - | - | 1.000 | 0.302*** | 0.372*** |
| Educational Value (EV) | - | - | - | - | - | 1.000 | 0.319*** |
| Reflective Feedback (RF) | - | - | - | - | - | - | 1.000 |

Note: N = 452. I = Interest; E = Engagement; ER = Emotional Response; C = Comprehension Ability; AE = Audiovisual Experience; EV = Educational Value; RF = Reflective Feedback. All correlations were statistically significant, ***p < .001. Values are standardised correlations among the latent constructs estimated in the measurement model (Supplementary S3). Dashes indicate values redundant with the upper-triangle entries.

**Supplementary S5. Statistical comparison of CK performance across T0, T1, and T2**

This supplement reports the statistical analyses of changes in Content Knowledge (CK) across T0, T1, and T2 based on individually linked responses (N = 452). Total CK scores (0–7) were analysed using one-way repeated-measures ANOVA followed by paired-samples t-tests. Descriptive statistics and pairwise comparisons are presented below.

**Table S4.Paired comparisons of Content Knowledge (CK) performance across T0, T1, and T2**

| Comparison | t | df | p | Cohen's d |
| --- | --- | --- | --- | --- |
| T0 vs. T1 | 34.47 | 451 | < .001 | 1.62 |
| T1 vs. T2 | 7.74 | 451 | < .001 | 0.36 |
| T0 vs. T2 | 41.61 | 451 | < .001 | 1.96 |

Note. Statistical comparisons were conducted using individually linked person-level CK responses at T0, T1, and T2 (N = 452; ID-matched across occasions). The paired comparisons in Table S4 are follow-up tests to the omnibus repeated-measures ANOVA reported above. All three pairwise comparisons were statistically significant. The T0-to-T1 gain reflected a very large effect (d = 1.62), consistent with substantial knowledge encoding during initial viewing. The T1-to-T2 comparison, although smaller in magnitude (d = 0.36), was also statistically reliable, indicating that CK continued to improve modestly over the two-week interval rather than reaching a complete plateau. The T0-to-T2 comparison reflected the largest cumulative effect (d = 1.96), confirming that gains were durable over the follow-up period.

**Supplementary S6. Descriptive statistics for main study variables**

This supplement reports descriptive statistics (mean, SD, and range) for the study variables used in the preliminary regression model (N = 452), including the composite experiential-dimension scores, Place-based Sustainability Stewardship Intention (PESI), and Content Knowledge (CK). CK scores were based on the answer key provided in Supplementary S2 and were obtained at the same T1 (immediate post-viewing) assessment as the experiential dimensions and PESI.

**Table S5.Descriptive statistics for main study variables**

| Variable | M | SD | Range |
| --- | --- | --- | --- |
| Content Knowledge (CK, out of 7) | 4.46 | 1.23 | 0-7 |
| Interest (I) | 3.49 | 0.88 | 1-5 |
| Comprehension (C) | 3.42 | 1.05 | 1-5 |
| Emotional Response (ER) | 3.43 | 0.96 | 1-5 |
| Educational Value (EV) | 3.54 | 0.88 | 1-5 |
| Engagement (E) | 3.17 | 0.94 | 1-5 |
| Audiovisual Experience (AE) | 3.33 | 0.82 | 1-5 |
| Reflective Feedback (RF) | 3.07 | 1.08 | 1-5 |
| PESI | 3.26 | 1.13 | 1-5 |

Note: All composite scores are means of a five-point Likert scale (1 = strongly disagree, 5 = strongly agree), N = 452. CK is scored as the number of correct responses out of seven dichotomous items (see Supplementary S2 for the answer key) and was calculated from the T1 (immediate post-viewing) assessment of the same participant sample (N = 452).

**Supplementary S7. Full preliminary regression table (PESI on experiential dimensions and grade level)**

This supplement reports the complete coefficient table for the preliminary multiple regression summarized at the start of Section 4, addressing calls for fuller regression reporting. Grade level was entered as two dummy variables (Grade_2, Grade_3), with Grade_1 (Years 1-2) as the reference category; Grade_2 corresponds to Years 3-4 and Grade_3 to Years 5-6. Model summary: R = 0.651, R² = 0.424, adjusted R² = 0.411, SE = 0.87.

**Table S6.Full regression coefficients: PESI on experiential dimensions and grade level**

| Predictor | B | SE | Beta | t | p | VIF |
| --- | --- | --- | --- | --- | --- | --- |
| Constant | -0.851 | 0.364 | - | -2.336 | .020 | - |
| CK | 0.000 | 0.133 | 0.000 | -0.002 | .998 | 1.037 |
| Interest (I) | 0.241 | 0.054 | 0.187 | 4.444 | <.001 | 1.358 |
| Comprehension (C) | 0.165 | 0.044 | 0.153 | 3.744 | <.001 | 1.278 |
| Emotional Response (ER) | 0.135 | 0.053 | 0.114 | 2.569 | .011 | 1.516 |
| Educational Value (EV) | 0.159 | 0.051 | 0.124 | 3.111 | .002 | 1.215 |
| Engagement (E) | 0.199 | 0.051 | 0.165 | 3.890 | <.001 | 1.372 |
| Audiovisual Experience (AE) | 0.201 | 0.057 | 0.145 | 3.546 | <.001 | 1.288 |
| Reflective Feedback (RF) | 0.056 | 0.044 | 0.053 | 1.268 | .206 | 1.345 |
| Grade_2 (Years 3-4 vs. 1-2) | 0.190 | 0.107 | 0.080 | 1.784 | .075 | 1.531 |
| Grade_3 (Years 5-6 vs. 1-2) | 0.426 | 0.104 | 0.179 | 4.091 | <.001 | 1.465 |

Note: Dependent variable: PESI. N = 452. All VIF values < 2, indicating no problematic multicollinearity.

**Supplementary S8. Follow-up study on extraneous cognitive load: descriptive statistics, correlations, and regression results**

This supplement addresses the reviewer's requests (Comments 14 and 15) to provide additional evidence clarifying the negative associations of Audiovisual Experience and Educational Value with Content Knowledge (CK) observed in the primary structural model. A supplementary session was conducted two weeks after the main survey with the same cohort (N = 452). Students completed a six-item cognitive-load scale (three extraneous-load items, two intrinsic-load items, and one mental-effort item) and then repeated the seven-item CK test. Descriptive statistics, the full correlation matrix, and univariate and multivariate regression results are reported in Tables S7-S10 below.

**Table S7. Descriptive statistics for the follow-up cognitive-load study variables**

| Variable | Items | M | SD | Range |
| --- | --- | --- | --- | --- |
| Content Knowledge (CK) | 7 | 5.10 | 1.31 | 0-7 |
| Extraneous Load (EL) | 3 | 3.57 | 0.53 | 1-5 |
| Intrinsic Load (IL) | 2 | 2.91 | 0.48 | 1-5 |
| Mental Effort (ME) | 1 | 3.40 | 0.88 | 1-5 |
| Audiovisual Experience (AE) | 3 | 3.39 | 0.44 | 1-5 |
| Educational Value (EV) | 3 | 4.36 | 0.38 | 1-5 |

Note: N = 452. CK is scored as number correct out of 7 dichotomous items. Extraneous Load, Intrinsic Load, Mental Effort, Audiovisual Experience, and Educational Value were rated on 5-point Likert scales (1 = strongly disagree, 5 = strongly agree).

**Table S8. Pearson correlation matrix for the follow-up cognitive-load study variables**

| Variable | CK | EL | IL | ME | AE | EV |
| --- | --- | --- | --- | --- | --- | --- |
| CK | 1.000 | -0.684*** | 0.090 | 0.041 | -0.264*** | 0.132 |
| EL | -0.684*** | 1.000 | -0.009 | -0.044 | 0.377*** | -0.186*** |
| IL | 0.090 | -0.009 | 1.000 | -0.015 | 0.088 | -0.004 |
| ME | 0.041 | -0.044 | -0.015 | 1.000 | -0.045 | 0.082 |
| AE | -0.264*** | 0.377*** | 0.088 | -0.045 | 1.000 | -0.064 |
| EV | 0.132 | -0.186*** | -0.004 | 0.082 | -0.064 | 1.000 |

Note: N = 452. CK = Content Knowledge; EL = Extraneous Load; IL = Intrinsic Load; ME = Mental Effort; AE = Audiovisual Experience; EV = Educational Value. ***p < .001. The correlation between CK and EV (r = 0.132) corresponds to p = .005 as reported in the main text but is shown here without an asterisk, consistent with the original source table.

**Table S9. Univariate regression models predicting Content Knowledge**

| Predictor | B | R² | p |
| --- | --- | --- | --- |
| Extraneous Load | -1.702 | 0.468 | < .001 |
| Audiovisual Experience | -0.785 | 0.070 | < .001 |
| Educational Value | 0.453 | 0.018 | .005 |

Note: N = 452. Each row reports a separate simple linear regression with Content Knowledge as the dependent variable. Audiovisual Experience alone accounted for 7.0% of the variance in Content Knowledge; this association became non-significant once Extraneous Load was included in the multivariate model (Table S10), consistent with a mediation-like pattern in which Extraneous Load carries the negative association between Audiovisual Experience and Content Knowledge.

**Table S10. Multivariate regression model predicting Content Knowledge from Extraneous Load, Audiovisual Experience, and Educational Value**

| Predictor | B | t | p |
| --- | --- | --- | --- |
| Extraneous Load | -1.693 | -18.23 | < .001 |
| Audiovisual Experience | -0.022 | -0.16 | .872 |
| Educational Value | 0.020 | 0.11 | .914 |

Note: N = 452. Model R² = 0.468, F = 132.45, p < .001. Extraneous Load was the only statistically significant predictor; the associations of Audiovisual Experience and Educational Value with Content Knowledge were no longer significant once Extraneous Load was included.
